# Cystic Echinococcosis: A Rare Case of Brain Localization

**Published:** 2017

**Authors:** Ali BARADAN BAGHERI, Mohammad ZIBAEI, Mehdi TAYEBI ARASTEH

**Affiliations:** 1. Dept. of Surgery, Shahid Madani Hospital, Alborz University of Medical Sciences, Karaj, Iran; 2. Dept. of Parasitology and Mycology, School of Medicine, Alborz University of Medical Sciences, Karaj, Iran; 3. Dept. of Anesthesiology, Shahid Bahonar Hospital, Alborz University of Medical Sciences, Karaj, Iran

**Keywords:** Echinococcosis, Hydatid cyst, Brain, Surgery

## Abstract

Although Hydatid disease eradicated in many countries, it is still widespread in communities where agriculture is dominant. Cystic hydatidosis is significant public health problem in the regions with endemic echinococcosis. The hydatid cysts tend to form mostly in the liver or lung. Brain involvement is very rare. In the present report, we describe magnetic resonance imaging findings in an 18-yr-old male with cerebral echinococcosis, in Shahid Madani Hospital, Karaj, Iran in 2015. The patient, presented with headache, hemiparesis, impairment of speech, vomiting, and nausea. Computed tomography, magnetic resonance imaging, and surgical exploration proved a cyst in the superior portion of left temporal lobe. Pathological examination showed it to be a solitary primary cerebral hydatid cyst.

## Introduction

Cystic echinococcosis, a chronic disease caused by the larval form of the tapeworm Echinococcus granulosus, is one of the most important helminth-associated zoonoses globally (1). Hydatid disease is a cosmopolitan zoonosis, with endemic area especially in South America, South Europe, New Zealand, and Middle East (2). Iran is an important endemic focus of human hydatid disease and cystic hydatidosis cases have been reported from different parts of medical centers. (3). The most common locations for cystic echinococcosis are the liver, followed by the lungs. However, other organs can be affected including bones, orbits and brain (4). Due to nonspecific clinical signs, the definitive diagnosis is based on serological, imaging, and histological findings (5).

In the current article, we report a case of cerebral echinococcosis, presented with headache, vomiting, and problems with speech.

## Case Report

**Fig. 1:**
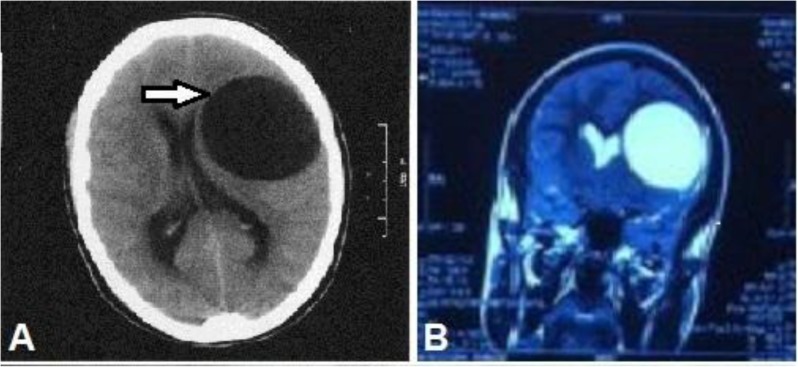
(A) Cerebral CT scan, cystic mass that measured 55 × 57 mm in the superior portion of left temporal lobe (B) Magnetic resonance imaging of brain that showing a large cyst lesion in the superior portion of left temporal lobe

In 2015, an 18-yr-old male presented to Shahid Madani Hospital, Karaj, Iran with a history of nausea, vomiting, and feeling of muscular weakness for 3 months. In addition, he complained of disorder in speech, weight loss, without any fever. Physical examination revealed disoriented with bilateral papilledema and hemiparesis on the right side of the body. Cerebral magnetic resonance (MR) imaging and computed tomography (CT) scan demonstrated a 55×57 mm huge cystic mass lesion in superior portion of left temporal lobe (Fig. 1).

**Fig. 2:**
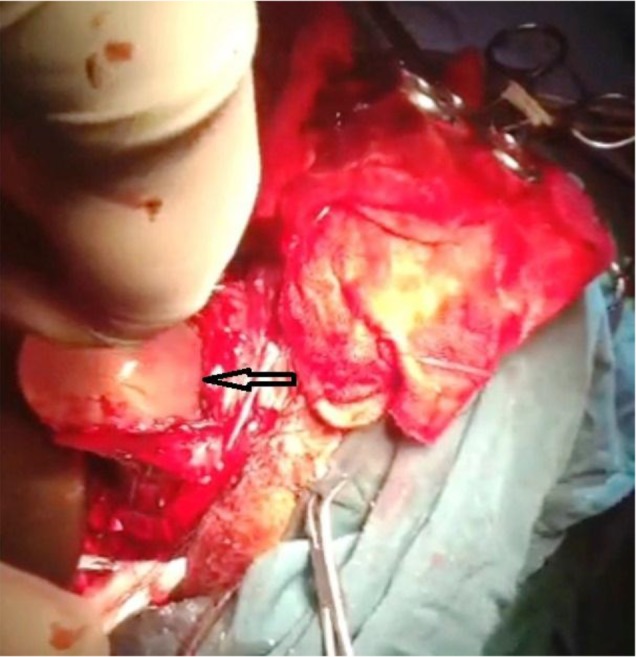
Cystic lesion the superior portion of left temporal lobe that includes hydatid cyst

Complete mass effect and midline shift as active hydrocephalus was visualized. Sign of ICP rising such as bilateral papilledema with early sign of subfascial herniation was notable (Fig. 2). The complete blood count showed a leukocyte count of 13200 *μ*L with eosinophilia of 4%. The results of biochemistry tests were as follows: Urea (25.0 mg/dL), Creatinine (1.1 mg/dL), and Uric Acid (5.1 mg/dL) (Table 1).

**Table 1:** The patient's laboratory test results

**Indicator**	**The patient's values**	**Normal**
White blood count (×1000/μl)	13.5	4.0–10.0
Red blood count (×10^6^/μl)	4.53	4.3–5.5
Eosinophil (%)	4	1–4
Neutrophil (%)	76.8	50.0–70.0
Hemoglubin concentration (g/dL)	11.8	13.0–17.5
Hematocrit (%)	34.3	40.0–52.0
Platelet (×1000/μl)	188	140.0–440.0
Fast Blood Glucose (mg/dL)	122	70.0–110.0
Urea (mg/dL)	25	18.0–45.0
Creatinine (mg/dL)	1.1	0.7–1.4
Uric Acid (mg/dL)	5.1	1.6–8.2
Total Cholesterol (mg/dL)	159	Up to 200
Total Triglyceride (mg/dL)	98	Up to 200

**Fig. 3:**
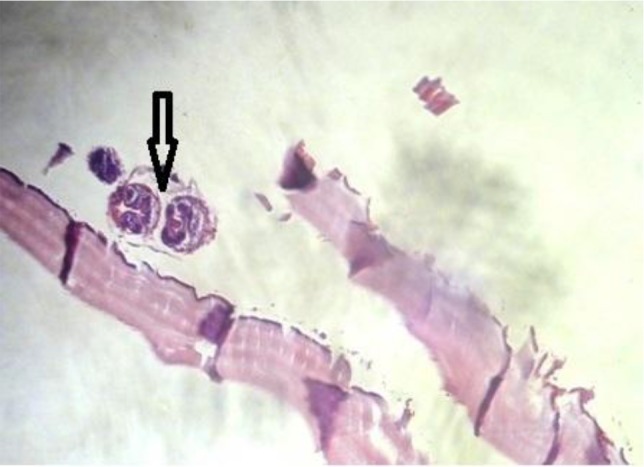
Histopathology of cerebral lesion, Cystic mass with protoscoleces is seen (Magnification: ×400)

In the pathologic examination of the specimen, irregular laminated layers or protoscoleces were noticed (Fig. 3).

After operation, the patient received albendazole (10 mg/kg/daily) in a course of 5 months (three weeks treatment separated by intervals a week).

Informed consent was taken from the patient.

## Discussion

Hydatid cysts often affect the lung and liver but rarely involves other organs such as the brain, so that more than one organ has been reported to be involved in 20%–30% of cases (6). Cystic echinococcosis involvement of the brain is an extremely rare condition even in endemic areas including Middle East, Mediterranean countries, South America, North Africa and Australia (7). Primary cysts are formed as a result of direct infestation of the larvae in the brain without demonstrating involvement of the most common ones are reported to be headache, papilledema, nausea, and vomiting. Any symptoms due to increased intracranial pressure can be seen (8). According to the size and location of the lesion, focal signs such as ambulate, convulsion, and hemiparesis can be seen. (9). MR imaging and CT scans show a well-defined oval cystic mass a low-intensity rim. The lesion typically shows no contrast enhancement, and calcification, usually peripheral, are rare.

Diagnosis of cerebral hydatid disease has been greatly facilitated with MR spectroscopy and albeit experimental. In a report, three cases of cerebral hydatidosis have been related to lactate, acetate, and succinate peaks, which surround with edema and increasing of choline and mannitol (10). Serological examinations have the problems of low diagnostic sensitivity, specify, and have only limited use (11).

The treatment of choice for hydatid cysts of brain is surgical excision. One of the methods of interest in the brain hydatid cyst surgery is Dowling-Orlando technique. In this method, the cyst can be released by lowering the head of the operating table and injection a tepid saline solution between the cyst and parenchymal tissue that surrounds the brain. Thus, the adhesions cyst wall to surrounding tissue is minimized. (12). Medical therapy is also important in intracranial hydatidosis involving use of benzimidazole carbonate derivates, such as albendazole and mebendazole. In comparison, albendazole is more effective than mebendazole and treatment should be continued for a few months (13).

## Conclusion

Although echinococcosis is endemic in Iran, only a few patients were reported to have had hydatid cyst in the brain. Cross-sectional imaging is crucial in differentiating hydatid disease from malignant lesions and this entity should be included in the differential diagnosis, especially in countries where the disease is endemic.
